# Increased On-Target Rate and Risk of Concatemerization after CRISPR-Enhanced Targeting in ES Cells

**DOI:** 10.3390/genes14020401

**Published:** 2023-02-03

**Authors:** Valérie Erbs, Romain Lorentz, Benjamin Eisenman, Laurence Schaeffer, Laurence Luppi, Loic Lindner, Yann Hérault, Guillaume Pavlovic, Marie Wattenhofer-Donzé, Marie-Christine Birling

**Affiliations:** 1 CNRS, INSERM, Université de Strasbourg, CELPHEDIA, PHENOMIN-Institut Clinique de la Souris (ICS), 1 rue Laurent Fries, 67404 Illkirch Graffenstaden, France; 2 CNRS, INSERM, Université de Strasbourg, Institut de Génétique et de Biologie Moléculaire et Cellulaire (IGBMC), Department of Translational Medicine and Neurogenetics, 1 rue Laurent Fries, 67404 Illkirch Graffenstaden, France

**Keywords:** gene targeting, double-strand break, embryonic stem cells, genome editing, CRISPR/Cas9, homologous recombination, reproducibility

## Abstract

The French mouse clinic (Institut Clinique de la Souris; ICS) has produced more than 2000 targeting vectors for ‘à la carte’ mutagenesis in C57BL/6N mice. Although most of the vectors were used successfully for homologous recombination in murine embryonic stem cells (ESCs), a few have failed to target a specific locus after several attempts. We show here that co-electroporation of a CRISPR plasmid with the same targeting construct as the one that failed previously allows the systematic achievement of positive clones. A careful validation of these clones is, however, necessary as a significant number of clones (but not all) show a concatemerization of the targeting plasmid at the locus. A detailed Southern blot analysis permitted characterization of the nature of these events as standard long-range 5′ and 3′ PCRs were not able to distinguish between correct and incorrect alleles. We show that a simple and inexpensive PCR performed prior to ESC amplification allows detection and elimination of those clones with concatemers. Finally, although we only tested murine ESCs, our results highlight the risk of mis-validation of any genetically modified cell line (such as established lines, induced pluripotent stem cells or those used for ex vivo gene therapy) that combines the use of CRISPR/Cas9 and a circular double-stranded donor. We strongly advise the CRISPR community to perform a Southern blot with internal probes when using CRISPR to enhance homologous recombination in any cell type, including fertilized oocytes.

## 1. Introduction

The replacement or insertion of DNA regions of more than a few kilobases within the murine genome is routinely achieved to generate, for example, knock-in or knock-out genetically altered (GA) mice. However, the frequency of homologous recombination between the targeting vector (donor DNA) and the target gene is variable depending on the locus. In some cases, the homologous recombination (HR) frequency can be very low. In this context, for some loci, CRISPR/Cas9 genome editing directly in the embryo will require a high number of embryos to obtain the GA line (for example, see [1]). In embryonic stem cells (ESCs), some loci will also require the screening of a large number of independent ESC clones regardless of the different lines used and their genetic background. Because CRISPR/Cas-induced double-strand breaks boost the frequency of homologous recombination [2] and allow highly efficient recombination with targeting vectors containing minimal homology with the endogenous locus [3,4], it holds the promise of overcoming these barriers. CRISPR-assisted targeting in stem cells has thus been successfully employed, and there are a number of reports displaying its effectiveness [5,6,7]. Biallelic targeting and multiplexed targeting have also become possible [8,9], setting the stage for substantial improvements in mutant production. 

To overcome targeting failures in the International Knockout Mouse Consortium (IKMC), Schick and collaborators [10] successfully used a dual nickase strategy, with Cas9 (D10A) generating a single strand break on both antiparallel strands and induced repair by homologous recombination. These constructs have a short-deleted region near the 3′ loxP of the IKMC conditional targeting vectors in which the single guide RNAs (sgRNAs) can be designed. Nevertheless, most of the targeting constructs do not contain genomic sequence deletion in order to minimize disruption of the mouse genome and thus limit the risk of generating hypomorphic alleles [11].

However, CRISPR-assisted homologous recombination remains unexamined on a large scale in mouse ESCs. We therefore chose to investigate whether CRISPR could enable efficient targeting at a large number of genomic locations that were previously inaccessible by conventional targeting. We also examined the recombined locus very closely by performing Southern blotting with internal and external probes (inside or outside the region homologous to the targeting vector, respectively). We showed that unexpected/unusual recombination events occur. In a standard validation pipeline of a facility such as the ICS, these events can easily be detectable by a Southern blot analysis of the genomic DNA using an internal probe (i.e., a probe located in the targeting construct). Other techniques such as long-read nanopore sequencing [12], targeted locus amplification [13] or short-read whole genome sequencing would also allow for identification of such events, but the ease of implementation, the cost and the time required to obtain and analyze the results would not fit within our production pipeline; we, therefore, focused on Southern blotting. We have clearly shown that at least one supernumerary copy (or concatemer) of the whole plasmid is inserted at the locus. These concatemers can neither be detected by 5′ or 3′ long range PCR (LR-PCR) nor by Southern blotting with an external probe but are easily detected by a Southern blot analysis using an internal probe. 

## 2. Material and Methods

### 2.1. Selection of gRNAs and CRISPR/Cas9 Vector

**Figure 1:**
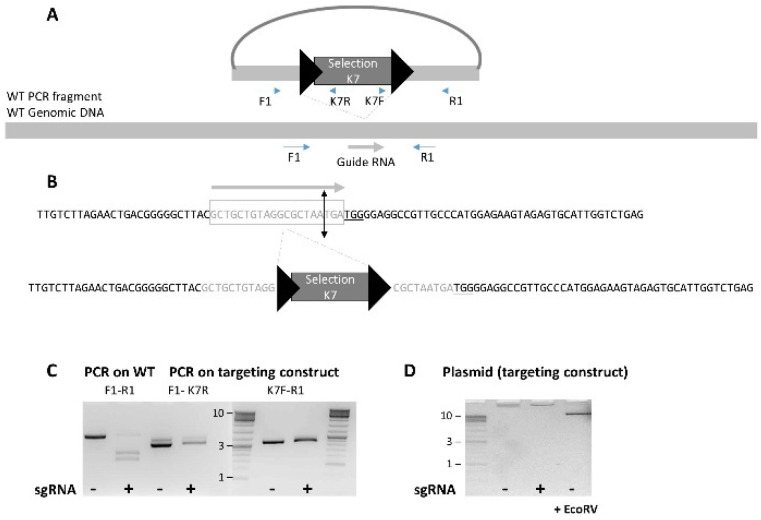
Example of a CRISPR design. (**A**) Scheme showing the position of the CRISPR-induced DSB relative to selection cassette. (**B**) Magnification of the sequences: a guide RNA was selected at the position of the insertion of the selection cassette so that the targeting construct would not be linearized. The PAM is underlined, and the 20 nt sequence recognized by the sgRNA is in light grey and surrounded by a rectangle. (**C**) PCR was performed on both the WT genomic DNA (F1-R1) and on the targeting construct in the region surrounding both LoxP sites (5′ F1-K7R and 3′ K7F-R1); the guide RNA was tested in the presence of the Cas9 protein on these PCR fragments (+); the PCR product alone is shown on the line indicated by a (−). Whereas a clear cut is observed on the WT PCR fragment, no DSB is observed on the PCR products from the targeting construct. (**D**) The whole targeting construct was run on a 1% agarose gel; (−) shows the pattern of the undigested plasmid. No linearization is observed in the presence of CRISPR (+), whereas a clear linearization (at the size of the targeting construct) is observed when the plasmid is digested with EcoRV (+EcoRV).

sgRNAs targeting the selection cassette insertion site were identified by directly inspecting the genomic sequence and were selected using the CRISPOR program (http://crispor.tefor.net/, accessed on 1 February 2023 [14]). The MIT specificity score was used to label the sgRNA (gR ‘MIT specifity score’). Each sgRNA was designed not to cut within the targeting vector but to cut only at the insertion site of the selection cassette (for an example, see Figure 1). Only guides with a score of >50 were selected. Each selected sgRNA was then cloned in the pX330 (#42230, Addgene [3]), and the resulting plasmid was sequenced and co-electroporated circular with the targeting construct (see Section 2.2.2).

### 2.2. Embryonic Stem Cell Techniques

#### 2.2.1. Embryonic Stem Cell Culture

PHENOMIN-ICS proprietary C57BL6/N ESCs (line name S3) were cultivated on mitotically arrested mouse fibroblasts (feeders) in KSR medium KNOCKOUT™ DMEM (Invitrogen cat N°. 10829-018); 15% KNOCKOUT™ SR (Invitrogen 10828-028); 40 µg/mL gentamicin, 0.1 mM β-mercaptoethanol (Sigma: M 7522), 1% GlutamaX (Invitrogen 35050-38), 1% non-essential amino acid (NEAA, Invitrogen 11140-050) and recombined LIF. Medium was renewed every day, and cells were generally trypsinized every two days (Trypsin 0.25% EDTA: Invitrogen 25200-072 diluted 1:2 in PBS). 

#### 2.2.2. Electroporation and Selection

Plasmids were electroporated using the Gene Pulser X cell Electroporation system (ref 165-2661, Bio-Rad, Hercules, CA, USA). In brief, 10 μg of circular targeting vector and 10 μg of CRISPR/Cas9 expressing plasmid (pX330, Addgene, Watertown, MA, USA) were combined to 5 × 10^6^ ESCs in a 4 mm electroporation cuvette and pulsed at 400 V, 125 µF. Cells were then distributed on two 10 cm plates with DR4 feeders (mouse embryonic fibroblasts derived from the Dnmt1tm3Jae Hprtb-m3 Tg(pPWL512hyg)1Ems/J line; [15]) in KSR medium. Twenty-four or 48 h after electroporation, medium was renewed with KSR medium containing either G418 (125–150 µg/mL) or hygromycin (125 µg/mL), respectively, depending on the selection cassette inserted in the targeting vector. This selection medium was renewed every day until clone picking (generally about 10 days after electroporation). Picked colonies were trypsinized and triplicated into 96-well plates. One plate served for the initial screen, the second plate was used to amplify the selected (LR-PCR positive) clones and the third plate was conserved as a back-up. Cells were allowed to grow again in 96-well plates containing feeders for about 4–5 days in KSR medium. 

#### 2.2.3. Cell Lysis

Plates were washed with PBS. Seventy-five µL of lysis buffer (ref 302-C Viagen) and 5 µL proteinase K (10 mg/mL) were added to each well. Plates were wrapped with foil lids and shaken slowly and incubated for 5 h to overnight on a plate shaker at 55 °C. The next day, the plates were shaken for a few minutes in order to homogenize the lysate. Eighty µL of H_2_O were added to each well to compensate for evaporation. Three wells (one with normal, one with high and one with lower cell content) were kept apart and served as controls for the PCR. In a 96-well plate, 1 µL of each ESC lysate was added to 5 µL of a solution of Tris HCl at 5 mM, and proteinase K was inactivated 5 min at 95 °C.

### 2.3. Embryonic Stem Cell Validation

#### 2.3.1. Long-Range PCR

##### Design of LR-PCR Genotyping Primers

The gene-specific LR-PCR genotyping primers used to identify clones were designed as follows: primers were chosen by examining the genomic sequence flanking the 5′ and 3′ targeting vector homology arms making sure to avoid repeated sequences.

The 3′ LR-PCR (binds in the vector 3′ sequence) was performed with a gene-specific reverse primer and a forward selection cassette universal primer (see Table 1 for universal primer sequences). 

The 5′ LR-PCR is also combined in a mixture with a universal reverse primer (Table 1) with a gene-specific forward primer.

##### Long-Range PCR Conditions

Screening reactions were performed with a combination of two DNA polymerases, Red Hot *Taq* polymerase (previously ABGENE-Ref. AB-0406/B, now special production by Thermofisher) and *Pwo* DNA polymerase (Roche). Genotyping was carried out on 96 well plates using 20 µL reaction volumes. In each well except the 3 last ones was placed: 2 µL universal or gene-specific forward primer (1.25 µM), 2 µL gene-specific or universal reverse primer (1.25 µM), 6 µL DNA lysate (corresponding to approximately 50 ng), 2 µL 10× buffer, 1.2 µL MgCl_2_ (25 mM), 0.4 µL dNTP (10 mM), 0.15 µL Red Hot *Taq* polymerase (5 U/µL), 0.02 µL *Pwo* DNA polymerase (5 U/µL) and 6.23 µL water. To validate each assay, as no positive control clone was available, the last three wells were kept for control PCR designed as follows: the internal universal primer was replaced with a gene-specific primer that was designed at the extremity of the most distal part of the homology arm. A screen can only be validated if at least one of these wells shows a PCR fragment at the expected size.

The cycling conditions were as follows: denaturation step of 5 min at 96 °C, then 30 cycles of (96 °C for 8 s, 60 °C for 10 s, 68 °C for 6 min) with an increase of 15 s at each cycle followed by 5 cycles of (96 °C for 8 s, 60 °C for 10 s, 68 °C for 8 min); to finish, a cycle of 10 min at 68 °C was applied.

#### 2.3.2. Southern Blotting

All Southern blots were performed using radioactively-labelled probes (α^32^P) following the protocol described in Codner et al. [16]. In our experience, radioactive probes give accurate results and are more sensitive than Dig-labelled probes on mouse genomic DNA.

## 3. Results

### 3.1. Outcome of the Use of CRISPR/Cas9

#### 3.1.1. Increased Homologous Recombination Frequency in the Presence of a Plasmid Expressing Cas9 and a Specific Guide RNA

The French mouse clinic (PHENOMIN-Institut Clinique de la Souris; ICS) has generated more than 2000 ‘à la carte’ mouse models by standard recombination and electroporation in ESCs [17,18,19,20]. The majority of these lines were generated with ‘*in house’* C57BL/6N-derived ES cells that are highly germ line competent. In our constructs, the average size of both 5′ and 3′ HR arms was 2.6 kb. In the absence of CRISPR, our constructs are always linearized before being electroporated, as targeting frequency with a circular plasmid has been shown to be much less efficient [21]

For 60% of gene targeting projects, the targeting efficiency was greater than 6.5% (more than 6 5′- and 3′-LR-PCR positive clones), and the screening of 93 ESC clones was sufficient to obtain at least two fully validated ES cell clones. For 90% of the projects, at least one positive clone was obtained through homologous recombination in C57BL/6N ES cells (targeting efficiency greater than 1.1%). In other words, only 15 out of our latest 144 gene targeting attempts (~10%) did not result in a positive clone when 93 clones were picked and analyzed. Our standard validation process includes a first (primary) screening by 5′ and 3′ LR-PCR. Then, after amplification of a maximum of 8 LR-PCR clones, a secondary screen is performed that includes a confirmation of the on-target events by the same 5′ and 3′ LR-PCR, additional LR-PCRs confirming the presence of important elements of the construct (LoxP, KI, etc.), followed by a careful Southern blot analysis with an internal and an external probe [16], as well as chromosome counting [22]. The large majority of the clones analyzed (98%) show only one band at the expected size when an internal probe is used for the analysis. When an additional band is observed, the size of this band is completely random and does not correspond to the digested plasmid size, suggesting the detection of an additional random integration of the targeting vector. 

For the majority of these targeting failures showing low homologous recombination rates, we had to screen more than 400 clones, but still 2–3% of our gene targeting projects (i.e., 20% of the projects with targeting efficiency below 1.1%) were reproducibly unsuccessful to produce a positive clone, even with (a) repeated electroporation attempt(s).

In an attempt to increase the frequency of homologous recombination at the target locus, we performed CRISPR-assisted electroporation. We used the same targeting constructs (no modifications, with the same homology arms) and co-electroporated them with a circular CRISPR/Cas9 expressing plasmid containing a specific guide RNA. The purpose of this CRISPR/Cas9 construct was to generate a double-strand break (DSB) at the site of insertion of the selection cassette (NeoR or HygroR cassette) that will activate the homologous recombination cellular repair pathway, allowing for correction using the targeting construct as the recombination template. 

We were able to find a specific guide RNA for all projects, even when the targeting construct was not designed to be used with CRISPR/Cas9. We checked that the selected sgRNA was not able to generate a DSB in the targeting vector but only at the site of insertion of the selection cassette (an example is illustrated in Figure 1). The fact that the guide RNA target sequence is not recreated in our targeting construct renders impossible an unwanted DSB [23].

Electroporation was performed with both targeting and CRISPR/Cas9 constructs as circular plasmids. We made the assumption that a CRISPR-mediated double-strand break would promote recombination at the locus and that a circular plasmid would have little choice but to integrate at the break rather than integrate randomly. Most electroporations were performed with 10 or 20 µg of each plasmid and gave successful results (fully validated positive clones). Lower concentrations were tested for two projects. For the first project, we decreased the concentration of both circular plasmids and tested three other concentrations: 5 µg + 5 µg, 2.5 µg + 2.5 µg and 1 µg + 1 µg of each plasmid. Positive clones were obtained for each concentration. The number of antibiotic resistant clones decreased with the plasmid concentrations, but the percentage of positive clone by LR-PCR PCR was similar. Respectively, 10 µg + 10 µg gave 72% positive clones; 5 µg + 5 µg: 84.9%; 2.5 µg + 2.5 µg: 85.4% and 1 µg +1 µg: 87.5% (see Table 2). For the second project, we tested only two concentrations of circular plasmids (10 µg + 10 µg and 1 µg + 1 µg), and we obtained positive clones for both conditions, but the percentage of positive clones dropped from 22.6% to 5.4% (see Table 2). 

#### 3.1.2. Linear Versus Circular Targeting Construct

**Figure 2:**
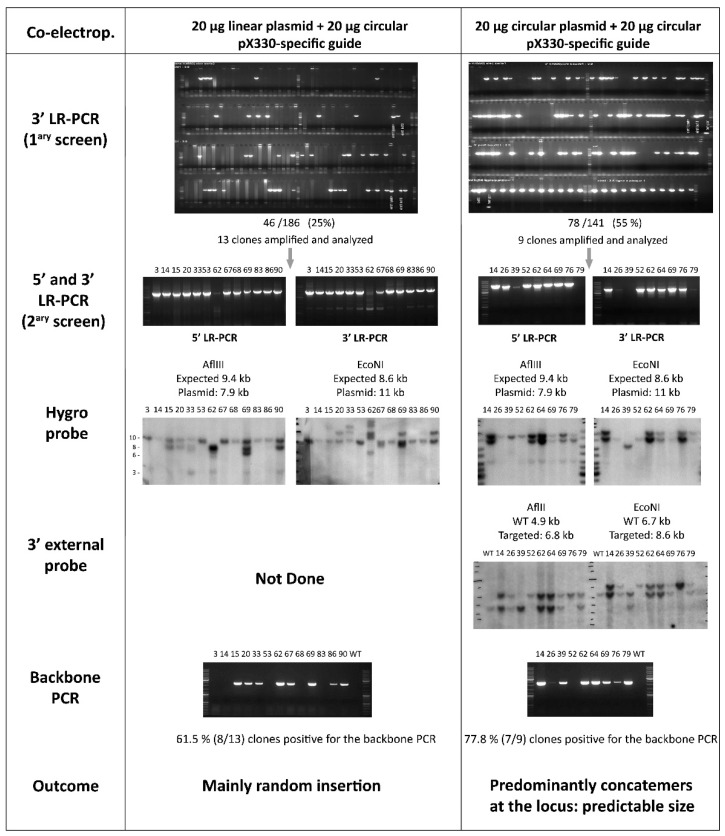
A construct that did not give any LR-PCR positive clones was co-electroporated linear (20 µg) and circular (20 µg) plasmids the same day in ESCs. The same CRISPR plasmid was used (20 µg) for both electroporation. Resistant clones were picked and subcloned, and ESC lysates were screened by 3′ LR-PCR (1^ary^ screen). Twenty five percent clones (46/186 clones screened) were found to be positive when the linearized construct was used; 55% clones (78/141) were positive when the construct was circular. Thirteen clones obtained with the linearized construct were amplified, and all were confirmed by 5′ and 3′ LR-PCR (clone 62 gave faint PCR products); similarly, nine clones obtained with the circular construct were amplified and eight were confirmed by both 5′ and 3′ LR-PCR. Amplified ESCs were analyzed by Southern blotting with an internal probe (Hygro probe). A single band at the correct size was expected, the expected sizes for each restriction digests are indicated (see Suppl. Figure S1 for detailed schemes). Five clones (3, 14, 53, 68 and 83) show a unique band for each digest when the linear plasmid was electroporated and two clones (26 and 62) when the circular plasmid was electroporated. Strikingly, the Southern blot pattern of the other (unvalidated) clones was very predictable when the targeting construct was used as circular and the size of the additional band corresponded to the complete plasmid fragment size (including the plasmid backbone). When the linearized construct was used, numerous additional insertions were observed. A Southern blot probe with a 3′ external probe (only performed on clones obtained with a circular construct) showed an expected pattern with a WT band and a targeted band as the expected size. Note that clone 52 could be homozygous (no WT band detected). All clones with an incorrect Southern blot pattern were also positive for a PCR (5′ backbone PCR) specific for the targeting construct backbone. A ‘backbone PCR’ performed between the plasmid backbone and the extremity of the 5′ HR arm permits easy recognition of the incorrect clones (see Figure 3 for the position of the PCR primers).

The use of a linear versus a circular targeting construct (co-electroporated with the circular CRISPR/Cas9 construct) was also assessed. Twenty micrograms of a linearized or circular construct were used on the same day to eliminate bias with regard to state of the ESCs. Figure 2 shows the PCRs that were performed to screen the HygroR clones (primary screen by 3′-LR-PCR,), as well as the 5′ and 3′ LR-PCR used to confirm the accurate recombination at the locus. The percentage of LR-PCR positive clones was higher when a circular plasmid was used (55%; 78/141 clones) compared to a linearized plasmid (25%; 46/186). Thirteen and nine clones, respectively, were amplified in order to analyze further some of these LR-PCR positive clones.

#### 3.1.3. An Unexpected and Specific Pattern

A Southern blot with an internal probe located in the HygroR selection cassette (Hygro probe) confirmed that, even if recombination occurred at the locus (i.e., positive 5′ and 3′ LR-PCR), additional insertion also occurred in some clones (Figure 2 and Suppl. Figure S1). Strikingly, for each restriction digest, an identical pattern was observed for most clones when the targeting construct was delivered in a circular form. Upon closer examination of the size of these bands, we noted that the additional band did correspond to the size of the plasmid when digested with the restriction enzyme (Figure 2 and Suppl. Figure S1). Importantly, these specific patterns were exclusively observed when CRISPR/Cas9 was added to improve the recombination efficiency. We have validated more than 1500 gene targeting events with an internal probe and have never observed this pattern without CRISPR/Cas9. For the sake of clarity of Figure 2, we only show two restriction patterns, but, in our standard validation scheme, we use four restriction enzymes and make sure that two of these confirm the 5′ and two the 3′ homologous recombination. For all four digests, the size of the additional band, when observed, corresponded to that of the digested plasmid.

**Figure 3:**
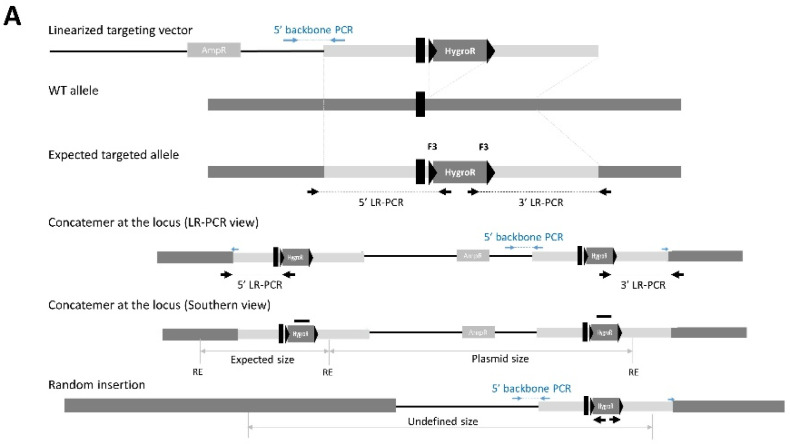

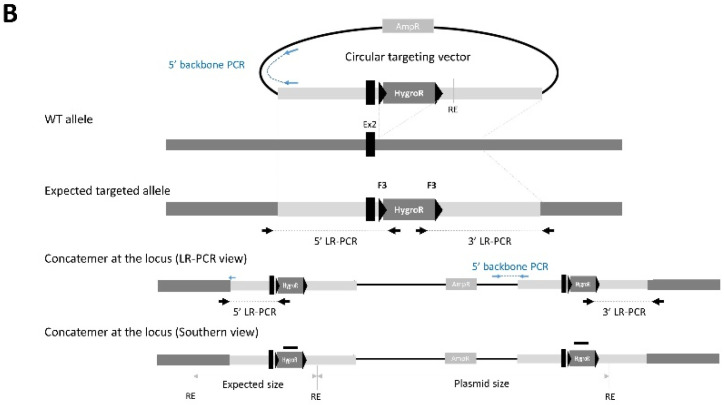
Scheme showing with the various alleles that are possible when a construct is electroporated in linear (**A**) or circular (**B**) form in the presence of a CRISPR/Cas9 construct. The position of the primers is shown. A scheme for a concatemer allele is shown here; both the LR-PCR view and Southern blot view (one restriction digest and one internal; Hygro probe, bold black line) are illustrated.

Simultaneously to the Hygro probe Southern blot, we performed a Southern blot with a 3′ external probe (for clones obtained with the circular plasmid only). To our surprise, the restriction pattern for all LR-PCR positive clones was exactly as expected. For clone 52, no wild-type (WT) band was observed, which may mean that this clone was targeted for both its alleles. We performed an additional PCR, specific for the backbone of the targeting construct, which showed that most of the LR-PCR positive clones also retained the vector backbone (Figure 2; backbone PCR; positions of the backbone PCR primers are indicated in Figure 3). Similar observations were made at the same time on different ongoing projects when using circular plasmids. All these observations lead us to hypothesize that concatemers at the locus could explain this very specific Southern blot pattern.

### 3.2. The Reduction of Homology Arm Size Does Not Affect Homologous Recombination Frequency

We compared two targeting constructs that were identical in all but the size of their homology arms. The first targeting vector, called the long arms (LA) vector, had a 5′ HR arm of 4.1 kb and a 3′ HR arm of 3.3 kb (i.e., classical HR arms for standard HR in ESCs); the second vector, called the short arms (SA) vector, had a 5′ HR arm of 0.41 kb and a 3′ HR of 0.52 kb.

Without a CRISPR/Cas9 expressing plasmid, we were not able to obtain any positive clones when electroporation was performed with the linearized LA vector (93 clones analyzed). We then performed, the same day, two new electroporations, both in the presence of a CRISPR expressing plasmid (20 µg), one with the circular LA vector (20 µg) and the other with the circular SA vector (20 µg). After screening the NeoR clones by 3′ LR-PCR, we obtained 88.7% positive clones with the LA construct and 78% with the SA construct. Five LR-PCR positive clones per condition were amplified and analyzed by Southern blotting with an internal probe, and we observed concatemers with both LA and SA constructs (1 in 5 with LA construct; 2 in 5 with SA construct). Similar observations were made by Schick et al. [10]. Our results suggest that long HR arms (3–5 kb) are not necessary for efficient ESC gene targeting in the presence of a CRISPR/Cas9 expressing plasmid.

### 3.3. Confirmation of On-Target Concatemer(s)

**Figure 4:**
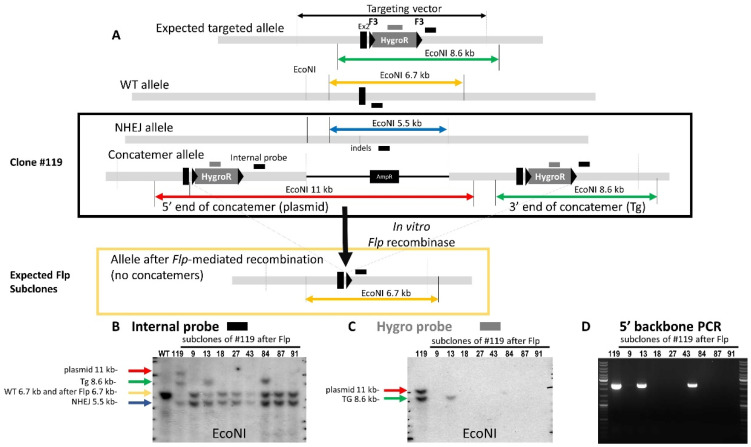
Evidence that concatemers occur at the locus. (**A**) Schematic drawing of the expected targeted, WT and clone #119 alleles (surrounded by a black rectangle). If concatemers at the locus occur, the action of the *Flp* recombinase, when optimal, should remove all the additional copies and leave only one F3 site. In order to visualize the allele (as the Hygro probe could no longer be used), we designed a new probe that recognized a 500 bps genomic sequence; this probe also recognized the targeting construct (in the 3′ homology arm). (**B**) Southern blot with an internal probe that recognizes both the genomic DNA and the targeting construct. The position of the WT probe is illustrated with a black bold feature in A. The WT line shows the expected WT fragment size. Line 119 shows the pattern of the paternal clone. This clone has an indel allele (blue arrow; 5.5 kb with an EcoNI digest) whose size differs from the WT allele (yellow arrow). A fragment that could not be distinguished from the WT size and which corresponds to the *Flp*-excised allele was observed in all of the subclones. The concatemer allele (corresponding to the size of the digested targeting vector; red arrow) was detected at 11 kb with the EcoNI digest. An EcoNI restriction fragment was observed at 8.6 kb in clone #119. Subclones 9, 18, 27, 43, 87 and 91 showed a *Flp* excision pattern with only one band at the expected size. No other band than the indel band could now be detected. A band at 8.6 kb could still be clearly detected in subclones 13 and 84, confirming that the excision of the additional copy was not complete. (**C**) The same clone and sub-clones were analyzed with the probe located in the hygromycin-resistance cassette. The typical concatemer Southern blot pattern was observed on parental clone #119 (expected size shown with a green arrow; plasmid concatemer shown with a red arrow); all subclones had their hygroR cassette excised except sub-clone 13. *Flp*-mediated excision between all F3 sites was not complete. (**D**) The 5′ backbone PCR was still positive for both sub-clones 13 and 84, confirming the persistence of an incomplete Flp recombination event; this was observed with the internal probe for both clones and the hygro probe for clone 13.

Our Southern blot results with the internal probe strongly suggested that we observed concatemers at the target locus rather than random insertion at other locations in the mouse ESC genome. We therefore designed the following experiment to confirm these initial results: we assumed that if we had concatemers, the additional copy(ies) (at the locus) should be removed if we administered a *Flp* recombinase to a clone that had a selection cassette surrounded by F3 site-specific recombination sites. In this case, only one copy of the targeting construct should remain after *Flp* mediated excision. We chose clone #119, which had the typical ‘concatemer’ pattern when an internal (HygroR) probe was used for Southern blotting (Figure 4A,C). This clone was positive for both 5′ and 3′ LR-PCRs and had the advantage of having, in addition to the targeted allele (with concatemer), an edited allele in place of the WT allele (Figure 4A). We designed an additional internal probe (internal probe; located in the 5′ region of the 3′ homology arm; in black in Figure 4) in order to be able to visualize the WT, the targeted, the *Flp*-excised and the indel (edited) alleles, as well as potential random insertion events. The edited (indel) allele can be easily distinguished from the standard WT allele and the Flp-excised allele using an EcoNI digestion (Figure 4A). A pCAG-Flpo plasmid (*in-house* construct) was then electroporated in ESCs amplified from this #119 clone. Ninety-three ESC clones were picked and analyzed by PCR with primers (FlpF-FlpR) located on both sides of the F3 surrounded selection cassette (see Suppl. Figure S2). Twenty-three clones had the expected PCR fragment (FlpF-FlpR, 0.64 kb). The WT band observed on the subclones (FlpF-FlpR, 0.56 kb) belongs to the feeder cell DNA. Eight clones showing this pattern were amplified (one F3 site remaining, size expected 640 bps). After Southern blotting with the internal probe (Figure 4A,B), a fragment at 6.7 kb was observed after EcoNI digestion of the ESC genomic DNA (Figure 4B). This confirmed that recombination occurred between the F3 sites present in the concatemer and that no additional random insertion occurred somewhere else in the genome. Figure 4A shows the position of both probes as well as the position of the EcoNI restriction site. Sub-clones #9, #18, #27, #43, #87, #91 show the expected fragment at 6.7 kb, confirming that *Flp*-mediated excision leads to only one final F3 site, whereas the mother clone #119 shows a typical concatemer pattern with the internal (Figure 4B) and the Hygro (Figure 4C) probes. A remaining band at 8.6 kb is still observed in sub-clones #19 and #84, showing that recombination between all F3 sites was not completely effective in these clones. The backbone PCR also detects the incomplete *Flp*-mediated recombination events and shows that, even if excision of at least one copy of the F3-surrounded selection cassette occurred, the whole excision was not efficient (Figure 4D). 

## 4. Discussion

In this study, we have shown that CRISPR/Cas9 provides new possibilities for the generation of mutant ESCs by dramatically increasing homologous recombination rates. Schick and collaborators [10] showed that a CRISPR-directed DSB, obtained using Cas9 nickase and two pairs of sgRNAs, permitted the identification of positive clones in studies that had previously failed within the International Mouse Phenotyping Consortium ((IMPC) https://www.mousephenotype.org/). They found, as did we, that reducing the size of targeting vector arms (from 5 kb to 1 kb) did not impact the homologous recombination efficiency; they obtained correct gene targeting for 35 of 75 vectors with 5 kb long arms (47%) and for 111 of 162 vectors (62%) with shorter 1 kb arms. In the presence of a CRISPR/Cas9 vector, reducing the size of the homology arms thus appears to have minimal impact. This facilitates the construction of complex targeting vectors, whether for regions of homology that are difficult to amplify or for large transgenes/modifications that require integration into a plasmid.

However, the presence of concatemers at the locus was never discussed in mouse cells, nor were they analyzed extensively. The presence of head-to-tail concatemers was described in a study using zebrafish [24], in which a circular plasmid was microinjected with CRISPR/Cas9 mRNA. An important difference was that CRISPR/Cas9-mediated knock-in of DNA cassettes into the zebrafish genome at a very high rate was obtained by homology-independent DSB repair pathways. After co-injection of a donor plasmid with a short guide RNA (sgRNA) and Cas9 nuclease mRNA, concurrent cleavage of donor plasmid DNA and the selected chromosomal integration site resulted in efficient targeted integration of donor DNA.

Several reports have described concatemerization events, which are common when a linear donor DNA is used. In 2020, Skryabin and collaborators [25] described unwanted head-to-tail insertions of linear DNA templates (double-stranded or single-stranded DNA) when CRISPR/Cas9 genome editing was used on hybrid fertilized oocytes. It was not clear which mechanism, homology directed repair and/or non-homologous end joining, was involved. Similar to our findings, the authors noted that conventional PCR, in most cases, failed to identify these multiple insertion events, which prompted them to alert the scientific community to the existence of a high rate of false positive alleles. Interestingly, they also used Southern blotting to confirm the presence of concatemers in some F1 pups from a mosaic founder. Smirnov and Battulin [26] discussed the possible concatenation mechanisms that may occur in the first moment after DNA injection of a linear transgene molecule; they speculated as to how cooperation of DNA repair pathways creates a multicopy concatenated insert. One of the models of concatenation predicts that a concatemer is built from one or more circular copies through de novo amplification or other means. This model could be inferred from the high efficiency of concatenation, the notion of occasionally observed identical transgene–transgene amplification [27] and the fact that cells possess mechanisms for gene amplification [28,29,30].

In the early 1980s, Folger et al. [31,32] showed evidence for homologous recombination between plasmid DNA molecules that were microinjected into cultured mammalian cells. In contrast to the plasmids used in this study, those plasmids did not bear homologous arms, and their insertion was not targeted. The authors showed that, in cells receiving injections of only a few plasmid DNA molecules, the transformation frequency was 40-fold higher after injection of linear molecules than after injection of supercoiled molecules. By controlling the number of gene copies injected into a recipient cell, they could obtain transformants containing a single copy or as many as 50–100 copies of the selectable gene. Multiple copies of the transforming gene were not scattered throughout the host genome but were integrated as a concatemer at one or very few sites in the host chromosome, and the orientation of the gene copies within the concatemer was not random; rather, the copies were organized as tandem head-to-tail arrays. They were able to conclude that the head-to-tail concatemers were generated predominantly by homologous recombination and that these head-to-tail concatemers were found in transformants obtained by injecting either supercoiled or linear plasmid DNA. It is possible that the concatemers we observed in this study were generated by a similar mechanism, implying homologous recombination between supercoiled plasmid molecules. In this case, the site-specific DSB mediated by CRISPR would induce homologous recombination at the locus with new homologous recombination events between the on-target copy and a new circular construct. We have not studied in detail the mechanism, as we focused on how to eliminate these undesired events and to select only clones that were properly targeted. 

Other approaches have been validated for obtaining locus targeting; for example, Suzuki and collaborators [33] devised a homology-independent targeted integration (termed HITI) strategy based on CRISPR/Cas9 technology. They used a CRISPR circular construct with linear or minicircle donor DNA with one or two Cas9/gRNA target sequences. As we use the same targeting plasmid (with homology arms) that failed to give positive clones without CRISPR/Cas9, we did not investigate this approach. 

It is likely that the on-target concatemers observed in this study are a product of homologous recombination. The fact that very few off-site insertions are observed (as suggested by the high rate of LR-PCR positive clones) and that the circular donor DNA cannot be linearized by CRISPR (see Figure 1 and Section 3.1) implies a recombination event mediated by homologous recombination. However, we cannot fully rule out another mechanism (see the review of Smirnow and Battulin [26]). An extensive study would be required to gain a full understanding of the concatemerization mechanism observed here.

Herein, we provide evidence that many clones with concatemers at the target locus are observed after CRISPR/Cas9 expression. We want to warn the scientific community that such concatemerization events may be frequent and that such clones should be discarded to avoid improper targeting. For all the targeting project analyzed, the concatemer Southern blot pattern is identical for most of the clones and is completely predictable when the construct is provided in a circular form; the additional band always corresponds to the plasmid fragment size recognized by the probe. This predictable size is strongly suggestive of a head-to-tail concatemerization of the construct, as head-to-tail or tail-to-tail concatemers would lead to different fragment sizes, which cannot be predicted. The use of a circular donor plasmid dramatically reduces the common random insertion observed when a linear construct is used (see Figure 2), and the CRISPR enhances on-target recombination. Our results highlight the importance of in-depth validation of targeted alleles. 

To distinguish these improperly targeted clones from those that underwent a unique recombination event, we defined a simple PCR screen that consists of a 5′ and 3′ specific PCR between the backbone of the targeting construct and the extremities of the 5′ and 3′ HR arms. Using one primer specific to the targeting construct for both PCRs allows us to drastically diminish the PCR contamination that could occur if primers were exclusively located in the backbone. In addition, these PCRs (called backbone PCR here), can be performed on selected 5′ and 3′ LR-PCR-positive ESC lysates at the stage of the primary screen (before ESCs’ amplification) in order to discard the ‘concatemer’ clones before any additional validation. We show that concatemers at the target locus are the most frequent unwanted event (compared to random insertions) when a circular targeting construct is used, representing 20–80% of the clones validated by both 5′ and 3′ PCR. The frequency of concatemer clones varies enormously from one project to another and we are not able to explain this variability. Figure 3 is a schematic of the various possible alleles, showing the position of primers used for the LR- and backbone PCRs, as well as the fragment sizes expected from Southern blotting.

These ‘concatemer’ clones have a typical Southern blot pattern when an internal probe is used. A band at the expected restriction size is always accompanied by a band corresponding to the size of the digested plasmid (taking into account the presence of restriction sites in the plasmid and the position of the probe). We were able to confirm that concatemers were specifically located at the locus and not anywhere else in the genome (Figure 4 and Suppl. Figure S2). The intensity of the additional (concatemer) band, similar to that of the expected band, suggests that only one additional copy was inserted at the locus.

We observed similar types of concatemers in vivo when the CRISPR/Cas9 reagents were microinjected in the presence of a circular plasmid (one experiment, not shown here). The unique difference resided in the number of additional copies, which was higher and could be explained by a longer dividing time of the 1-cell embryo (1 day from 1 cell to 2 cell stage) versus a few hours in ESCs. This means that concatemerization is a common feature after CRISPR-mediated homologous recombination when a circular plasmid is used and is certainly not specific to ESCs.

The use of CRISPR in addition to a standard targeting construct allows for a very significant increase in the number of positive clones. So, even if concatemers occur, a careful validation of the ESC clones always results in microinjectable clones. It should be noted that we did not observe any difference in the germ line transmission competency of CRISPR-treated clones. As was the case in other studies, we observed an increase in the number of homozygous clones (both alleles targeted) using CRISPR. The interpretation of Southern blots should be reassessed, as frequent indels (NHEJ events) occur at the CRISPR DSB site on the untargeted allele. An indel can change the pattern of the Southern blot performed with an external probe, as the WT allele band can be missing and can be replaced by another band of undetermined size (see Figure 4 as an example). The absence of a WT allele and its replacement with an indel allele also makes loss-of-allele quantitative PCR impossible for use in screening, as a reduction of the WT copy number can no longer be correlated to proof of recombination at the locus.

The mechanism that leads to the on-target concatemerization at the locus is unclear, as is why it occurs so frequently when CRISPR/Cas9 is used. The fact that the targeting construct is provided in circular form certainly favors this repair event. Indeed, random insertion (out of the locus) occurs much more frequently when the targeting construct is provided in linear form. It is possible that homologous recombination between circular plasmids occurs as previously described by Folger and collaborators [31], but the reason why the addition of CRISPR favors these events when a circular plasmid is used remains unclear.

We also tested our droplet digital PCR protocol (ddPCR), which was validated for precise DNA quantification [11], to select the correctly recombined clones (without ‘concatemers’) but the accuracy of the quantification was not sufficient to give clear-cut results. This may be due to the fact that the ESCs are often mosaics and/or that the quality of the DNA lysates used for the primary ESC clone screening was not optimal for accurate quantification. Although ddPCR is a particularly useful method for accurate detection of a DNA copy number, another reason for our unclear results may be that tandem gene copies or sequence concatemers may not be correctly separated into droplets in ddPCR experiments, leading to an inaccurate copy number variant estimate [11]. In this particular case, the entirety of the concatemers was not correctly spread throughout the droplets (a number were trapped in some droplets) due to the physical proximity of the various concatemer copies. We are currently investigating optimization of these ddPCR experiments by performing enzymatic digestion of the genomic DNA prior to generating droplets [34]; however, partial digestion of the DNA target must be avoided and this seems to be difficult to avoid [35].

Concatemer alleles are not always to be discarded and can also be an advantage for model creation. For instance, when conditional expression (before Cre-mediated excision) is expected, such alleles can be driven towards the correct recombined allele after Cre-mediated excision of the supplementary copy. Likewise, one or more additional copies can lead to overexpression (in the case of a targeted transgene or a gene humanization) with the advantage of being at a known locus. It is important to be aware of the potential outcomes, to fully validate the alleles and to choose the one that is of interest.

Importantly, our targeting constructs do not contain a diphtheria toxin A (dTA) negative selection cassette. In theory, the presence of a dTA cassette in the targeting construct backbone should limit, if not avoid, concatemers, by killing ‘concatemer’ clones. We performed a test in which two identical constructs, in all respects except for the presence of the dTA in the backbone, were electroporated in the presence of CRISPR/Cas9 the same day. We fully characterized all the LR-PCR clones and ended up with eight fully validated positive clones when no dTA was in the plasmid backbone, whereas two clones were only fully validated when dTA was present in the backbone. We decided not to add dTA in the backbone of our constructs due to limitations of the plasmid size and because negative selection (dTA bears its own promoter) could result in transient expression before the insertion of the circular construct and could lead to the death of positive clones.

In conclusion, CRISPR greatly improves homologous recombination rates and can be used to rescue previously failed projects. It is important to fully validate the clones that are chosen for microinjection as concatemers are frequent events when using CRISPR. We have demonstrated that these concatemers occur at the locus and that they are the most frequent unwanted event when the targeting construct is provided in circular form. They are not detectable by 5′ and 3′ LR-PCR or by Southern blotting with an external probe but can easily be identified using specific backbone PCRs (one primer in the backbone and one primer at the extremity of the homology arm). Southern blotting with an internal probe allowed us to confirm the nature of the concatemer and emphasizes the importance of this technique for accurate validation of any knock-in allele in our validation pipeline. Finally, although we only tested murine ESCs, our results highlight the risk of mis-validation of any genetically modified cell line (such as established lines, iPS cell lines or those used for ex vivo gene therapy) that combines the use of CRISPR/Cas9 and a circular double-stranded donor.

We strongly advise the CRISPR community to perform Southern blotting with internal probes (alternatively, to use any other accurate estimation of the donor DNA copy number) when using CRISPR to enhance homologous recombination in any cell types, including fertilized oocytes.

## Figures and Tables

**Table 1:** sequence of universal primers currently used in the lab.

Selection Cassette	Universal Forward Primer for 3′ LR-PCR	Universal Reverse Primer for 5′ LR-PCR
Neomycin resistance (*NeoR*)	AGGGGCTCGCGCCAGCCGAACTGTT	GCGGCCGGAGAACCTGCGTGCAATC
Hygromycin resistance (*HygroR*)	CCGTCTGGACCGATGGCTGTGTAG	CTGCATCAGGTCGGAGACGCTGTCG

**Table 2:** Quantity of circular targeting construct and circular CRISPR construct.

	10 µg + 10 µg	5 µg + 5 µg	2.5 µg + 2.5 µg	1 µg + 1 µg
Project 1: Positive clones by long-range PCR (%)	67/93 (72%)	79/93 (84.9%)	41/48 (85.4%)	35/40 (87.5%)
Project 2: Positive clones by long-range PCR (%)	18/93 (19.4%)	ND	ND	5/93 (5.4)

## Data Availability

Not applicable.

## Supplementary Materials

The following supporting information can be downloaded at: https://www.mdpi.com/article/10.3390/genes14020401/s1, Figure S1: Scheme of the circular targeting construct and the various alleles, with the position of AflIII and EcoNI restriction sites. The positions of the three probes used for Southern blot analysis (Hygro probe in dark gray, internal probe in black and 3′ external probe in light gray) are indicated; Figure S2: *Flp*-mediated excision of the extra copy (concatemer). Clone 119 was submitted to Flp-mediated excision in ES cells. A. Scheme of the different alleles. PCR FlpF-FlpR was performed in order to follow the action of the Flp recombinase. B. Annotated sub-clones are clones for which two bands of the expected size were detected, one band corresponding to the WT allele amplified from the feeders (0.56 kb) and one band corresponding to the Flp-excised allele (0.64 kb). The initial clone is annotated 119. It should be noted that not all sub-clones committed a complete Flp excision, and some clones were mosaics. The band observed at ~1.2 kb must be a heteroduplex.
