# Supplementary material for: Increased On-Target Rate and Risk of Concatemerization after CRISPR-Enhanced Targeting in ES Cells
Source: Genes (Basel). 2023 Feb 3;14(2):401. doi: 10.3390/genes14020401 (PMC9957269; doi:10.3390/genes14020401)
Supplement: Supplementary file 1 [file genes-14-00401-s001.zip › genes-2175802-supplementary.pdf]

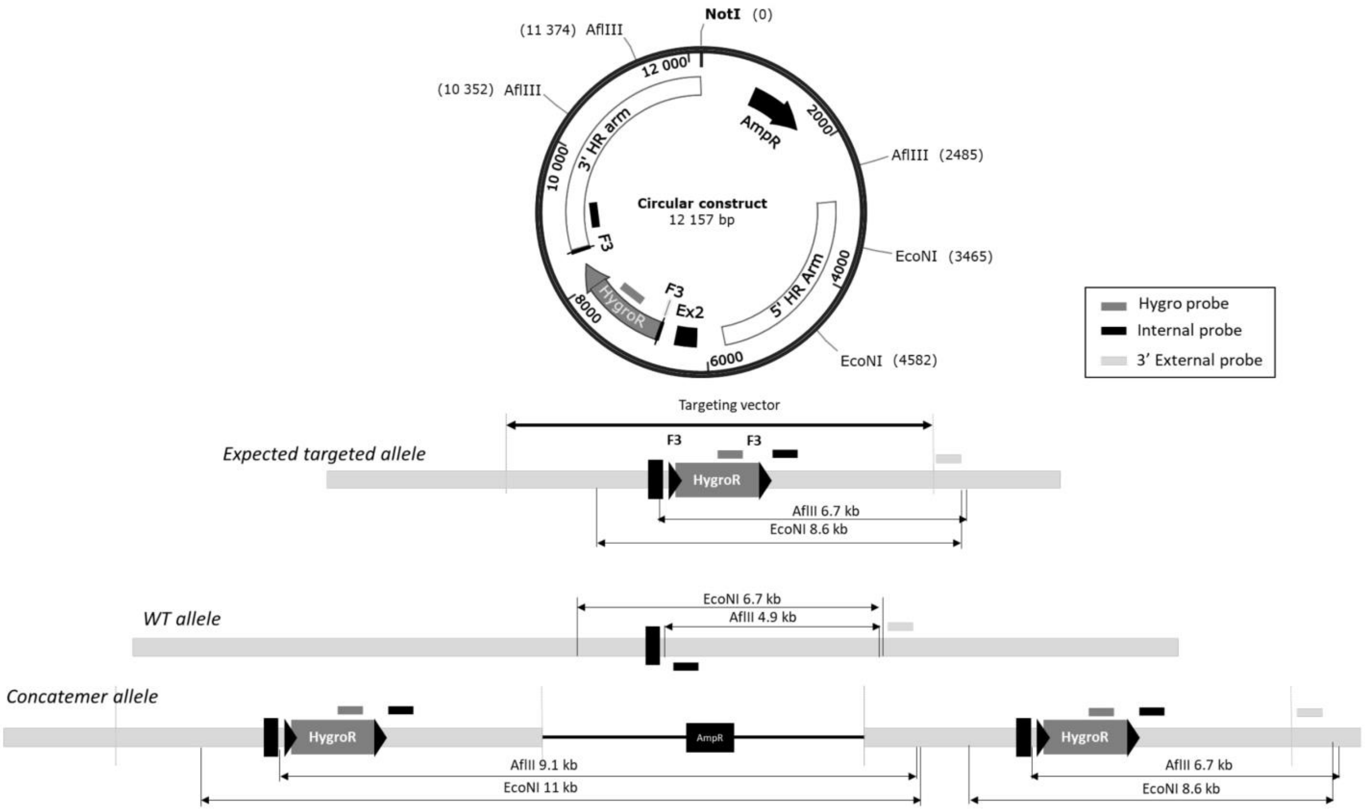

Figure S1. Scheme of the circular targeting construct and the various alleles, with the position of AflIII and EcoNI restriction sites. The positions of the three probes used for Southern blot analysis (Hygro probe in dark gray, internal probe in black and 3′ external probe in light gray) are indicated.

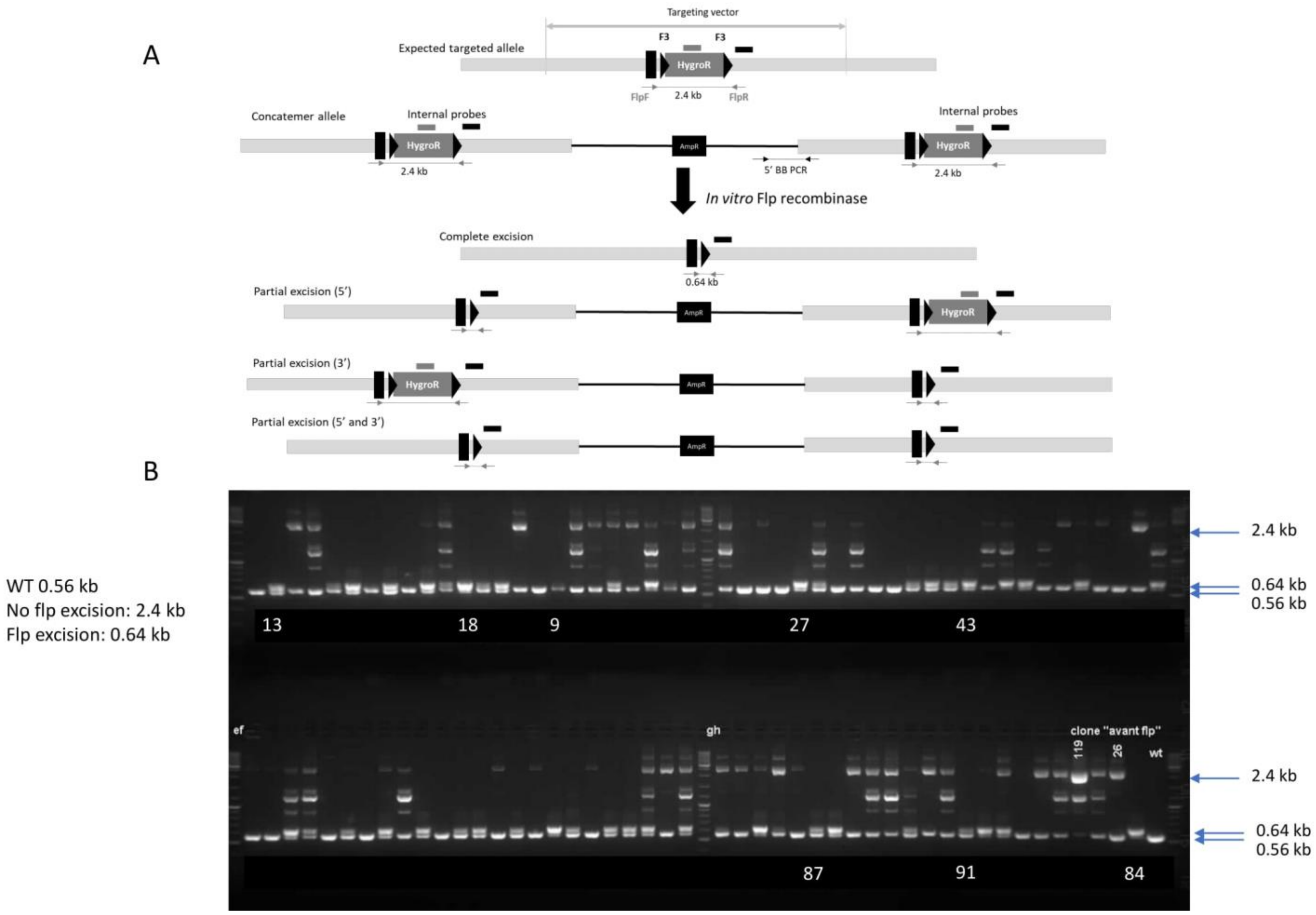

Figure S2. Flp mediated excision of the extra copy (concatemer). Clone 119 was submitted to Flp mediated excision in ES cells. A. Scheme of the different alleles. PCR FlpF-FlpR was performed in order to followed the action of the Flp recombinase. B. Annotated sub-clones are clones for which 2 bands of the expected size were detected, one band corresponding to the WT allele amplified from the feeders (0.56 kb) and one band corresponding to the Flp excised allele (0.64 kb). The initial clone is annotated 119. Noteworthy, not all subclones committed a complete Flp excision and some clones were mosaics. The band observed at ~1.2 kb must be a heteroduplex.
